# The impact of psychological distance on preferences for prenatal screening and diagnosis for chromosomal abnormalities: A hierarchical Bayes analysis of a discrete choice experiment

**DOI:** 10.1371/journal.pone.0324370

**Published:** 2025-05-23

**Authors:** Tima Mohammadi, Wei Zhang, Aslam H. Anis

**Affiliations:** 1 Centre for Advancing Health Outcomes, Providence Research, Vancouver, British Columbia, Canada; 2 School of Population and Public Health, University of British Columbia, Vancouver, British Columbia, Canada; 3 Faculty of Pharmaceutical Sciences, University of British Columbia, Vancouver, British Columbia, Canada; University of Brasilia: Universidade de Brasilia, BRAZIL

## Abstract

**Introduction:**

Hypothetical bias continues to be a primary challenge for stated preference methods. The source of hypothetical bias might be approached from the conceptual framework of “psychological distance.” By comparing the two samples of pregnant and non-pregnant women, this study aimed to investigate the impact of psychological distance from real-life choice on prenatal screening and diagnostic strategies preferences.

**Method:**

A discrete choice experiment was conducted among a sample of pregnant women and a sample of the general Canadian population. The attributes included: timing of the results, false-negative rate, false-positive rate, risk of miscarriage, and out-of-pocket cost. The dual response design, including forced and unforced choices, was used. Hierarchical Bayes modelling was employed to estimate part-worth utilities at the individual level. The relative importance scores of the attributes and willingness to pay for improvement in attributes were compared between pregnant and non-pregnant women. Using the individual-level preference weights, we also estimated the uptake rates for various scenarios and compared the two samples. We quantified hypothetical bias by comparing the real-world decision and predicted choices for different strategies for the pregnant and non-pregnant women samples.

**Results:**

A sample of 426 pregnant women was matched to 426 non-pregnant women from the general public sample. For pregnant women, the ability to detect chromosomal abnormalities was the most important attribute. For the matched sample of non-pregnant women, false-negative rate and risk of miscarriage were the most important attributes. In addition, pregnant women were willing to pay more for improvement in test characteristics and less sensitive to changes in strategy cost than non-pregnant women. The findings also showed a more significant difference between the actual and predicted choice among non-pregnant women.

**Conclusion:**

Our findings showed that although both groups valued safer and more accurate screening strategies, there was a difference in willingness to pay, sensitivity to cost, and predictive power of discrete choice experiment estimates between pregnant and non-pregnant women. This difference can be explained by their psychological distance from the decision. In conclusion, psychological distance impacts decision-making and can be identified as a source of hypothetical bias in measuring prenatal screening and diagnosis preferences.

## Introduction

Stated choice methods have been used widely in health to elicit value and provide information for policy decisions. But there has been an ongoing discussion on the extent to which learnings from stated choice studies can be generalized to real-life decisions. As such, one of the most important concerns about the use of stated preferences studies is the existence of hypothetical bias, which is the disparity in preferences in hypothetical and actual situations [1–4].

Some of this bias may be explained by adopting a conceptual framework of “psychological distance” [5]. According to this approach, an object or decision can be perceived as distant if it is not in the time (temporal distance) or space of the individual (spatial distance), it is considered as belonging to someone else (social distance) or, it is perceived with the low probability of occurrence (hypothetical distance) [6]. Construal level theory (CTL), a cognitive science theory, describes the impact of psychological distance on individual thinking about technologies or interventions [7–10]. According to CTL, an individual’s decision-making is either abstract or concrete, depending on how far the decision is perceived*.* Decisions and events that are part of the individuals’ direct experience are considered psychologically proximal when they have more detailed and reliable information about these decisions and events, and process and perceive them differently from the psychologically distant entities [9].

In many areas of health care, investigating public attitudes (potential users) can improve the decision-making process for interventions. Hence, it is important to know how preferences for certain health interventions are shaped by psychological distance (mostly hypothetical and temporal distance). This helps researchers better understand the source of the hypothetical bias and design effective mitigation techniques by reducing the respondents’ perceived psychological distance in stated preference studies. In health research, only a few previous studies have investigated the impact of psychological distance on perceived disease risk, choice, and behaviour [11–13].

This study aimed to explore whether psychological distance from the decision leads to a difference in preferences for prenatal screening strategies. Prenatal screening provides an important scenario and opportunity to study the impact of psychological distance on the decision-making process because non-pregnant women can be considered as the potential users of these strategies and their difference from the current users (pregnant women) is their distance to the real-life choice. By comparing the outcomes of a discrete choice experiment (DCE) conducted for samples of non-pregnant women and pregnant women, we were to evaluate the extent to which the distance from the real-life decision affected the preferences for a prenatal screening test. In other words, how people’s preferences changed when the hypothetical situation was changed to a more real one. Investigating the consistency of preferences, perspectives, and resulting hypothetical bias could provide essential evidence for policy decision-making in developing and improving prenatal screening programs in publicly funded health systems. To our knowledge, no previous studies have compared pregnant women’s and the public’s preferences for prenatal screening and diagnostic strategies. However, differences in preferences can impact choices and decision-making, and the values of both pregnant women and the general public must be considered in developing and implementing prenatal testing strategies. Therefore, it is important to investigate differences between preferences from a general population or societal perspective and the values that pregnant women place on aspects of prenatal screening. This study was to fill the gap in the literature.

## Materials and Methods

Good research practices for stated-preference studies were followed in conducting this study and analyzing the data [14–16]. Ethics approval for this study was obtained from the University of British Columbia Behavioural Research Ethics Board (H17-00591). Informed written consent was obtained from respondents.

### DCE development

Following recommendations for good research practices to conduct a DCE [15,17], we used a mixed-methods approach to develop the DCE attributes and questionnaire. This approach involved both quantitative and qualitative research components. Firstly, a literature review of studies on preferences for prenatal screening and diagnosis was conducted to gather information about potential attributes and levels. In addition, we conducted qualitative research. This qualitative study involved four in-depth, semi-structured focus groups with pregnant women and their partners/support people. Through this qualitative study, important factors such as the time of diagnosis, information on conditions tested, false positives, cost, the invasiveness of the test, and potential harm to women and babies were identified [18]. These themes showed the conceptual attributes important to participants’ decision-making for prenatal screening and diagnosis. After collecting a list of potential attributes and possible levels based on the literature review and qualitative study results, we consulted with an expert in medical genetics and prenatal screening about the face validity of these potential attributes and levels. This process led to the selection of five attributes and their corresponding levels: timing of the results, false negative rate, false positive rate, risk of miscarriage, and out-of-pocket cost. We also used the pre-screening risk of chromosomal conditions as a constant attribute.

The study design was similar to our previously published study estimating public preferences and willingness to pay (WTP) for prenatal screening and diagnostic strategies [19]. As described there, we applied experimental design techniques using SAS (SAS software version 9.4) [20] to generate optimal combinations of attributes and levels that respondents evaluated in the DCE. Searching for the maximum efficiency design was based on the D-efficiency measure given the constraints. Using this approach, 36 choice sets were generated and then were blocked into four versions of nine choice questions.

We applied the within-set dominated pairs test to assess respondents’ comprehension and consistency. Two fixed questions were added to each questionnaire version, and in these tasks, one of the options was worse than the other alternative in terms of all attributes. Respondents who chose the dominated strategy in both tests were considered to have failed the consistency test. In addition, using response time as a proxy for cognitive engagement, we set time criteria for the questionnaire based on the completion time for our study and the limits used in the literature [21]. We calculated the minimum amount of time for completing the questionnaire based on data from pilot testing (around 3 minutes). Therefore, we set three minutes as the minimum completion time to exclude responses from potentially inattentive people who completed the survey quickly and may not have given the DCE choice questions enough thought. We also wanted the respondents to complete the DCE as uninterruptedly as possible in order for them to be engaged, comprehend the concept of the DCE choice task, and understand the implicit trade-off between attributes. Hence, we assumed respondents who left the survey for longer than 24 hours might have provided less reliable responses. Therefore, those who completed the survey in less than three minutes, or more than 24 hours, or failed the two consistency checks were excluded from DCE data.

We employed a dual response choice experiment approach [22], in which respondents were first forced to choose among two prenatal screening and diagnostic strategies and then were asked to choose between their chosen strategy from the first stage and a “no screening” option (unforced choice). A sample of DCE questions for forced-choice and unforced choice is presented in S1 Fig. Background information on chromosomal conditions and prenatal screening and diagnosis was included in the questionnaire before the DCE choice tasks. Socio-demographic characteristics and experiences of pregnancy and prenatal screening were asked in the final section of the survey questionnaire. The full survey is included in S1 File.

After developing the DCE questionnaire, we conducted “think-aloud” interviews with 10 respondents as a qualitative pre-testing method to assess the questionnaire’s clarity and respondents’ interpretation. Further, as a quantitative test, two rounds of pilot testing were conducted to determine whether respondents could comprehend the attributes and choices and cognitively manage the number of choice tasks. The data obtained in two rounds of pilot testing was statistically analyzed separately and was not included in the main analyses. A total of 108 and 105 respondents completed the first and second rounds of the pilot testing, respectively. 101 (93%) and 95 (90%) respondents met the consistency and completion time requirements. Based on pilot testing data analysis, the estimated individual-level weights had expected signs and order, which showed that in general, respondents understood the DCE tasks and could manage the number of questions.

### Study sample

The online DCE survey participants (18 years and older) for this study were recruited by IPSOS, a global market research firm. Johnson and Orme’s rule of thumb [23] was used to determine the minimum sample size (S2 File), but we aimed for a much higher number for both samples to achieve sufficient statistical power to estimate preferences [24]. The first study sample of pregnant women was selected from a survey administered and completed by a group of pregnant women in Canada. The second study sample of non-pregnant women was selected from a survey administered and completed by a sample of Canadian general public members. The samples were collected in May and November of 2018. To evaluate the potential impact of excluding respondents based on time criteria or failing the inconsistency test on the study’s outcomes, we undertook a sensitivity analysis that included these respondents in the sample. This analysis was aimed at assessing the robustness of our findings by taking into account the criteria used for respondent exclusion.

Since the systematic differences between the characteristics of two groups of respondents (pregnant women vs. non-pregnant women) can be a potential confounder when comparing their preferences, we used a greedy matching algorithm to increase the validity of the comparison. We chose the sample of non-pregnant women and the sample of pregnant women who had similar baseline characteristics, i.e., age, sex, education, and whether having children using one-to-one matching. DCE response and demographic data from the matched samples of pregnant women and non-pregnant women were used for the analysis.

### Statistical analysis

In this study, the hierarchical Bayes (HB) [25,26] method has been used to effectively model preference heterogeneity and estimate individual-level part-worth utilities. By combining individual-specific choice data with the prior distributions of the parameters, HB provided a proper method to derive the individual-level utility weights. This approach enhances estimation precision and leads to more reliable individual-level estimates, especially with small sample sizes, by leveraging information from the entire study population. We used MATLAB, edition R2014 (The MathWorks, Inc., Natick, MA) for estimating the Bayesian hierarchical model. In the final model, 10,000 initial iterations were discarded as “burn-in,” and 20,000 iterations were used for the estimation. Since the draws from Gibbs samplings were correlated, to reduce the correlation, after convergence, every tenth draw was used, and the rest were discarded. The prior on the covariance matrix was inverted Wishart, and the prior on the random parameters was uninformative. Both forced and unforced data were analyzed. In the unforced model, a systematic preference for screening versus no screening was evaluated using an alternative-specific constant (ASC).

### Comparing two samples

After hierarchical Bayesian estimation of the mixed logit model, we computed the relative importance score of the attributes using the range between different levels within each attribute, which was scaled to 100 for both samples, and compared the importance of the attribute in relation to the other attributes presented in any of the two DCE studies. To account for the scale effect in the coefficients, we used the marginal rate of substitution (WTP) to compare the two samples’ preferences. Using the individual-level preference weight, we also estimated the probability of choosing different prenatal screening and diagnostic scenarios (the non-invasive prenatal test (NIPT), amniocentesis, chorionic villus sampling (CVS), and optimal case) for each respondent. Predicted uptake rates for various scenarios were computed and compared between the two samples. Furthermore, we compared the predicted choice of different prenatal screening strategies with the actual decision for each respondent in both samples to quantify hypothetical bias. All the analyses for comparing the two samples were based on the results of the unforced models (S3 File).

## Results

### Sample characteristics

In the first study sample, a total of 538 pregnant women completed the survey. Of them, 502 respondents passed the completion time criteria (more than 3 minutes and less than 24 hours), and then 432 were accepted after internal consistency checks. Six pregnant women could not be matched to one non-pregnant woman from the general public sample by age, sex, education and having children and thus were excluded. Our final sample included 426 pregnant women and 426 matched non-pregnant women. Most of the pregnant women were under 35 years old (78%) and mainly married or common-law (79%). Eighty-four percent of the sample were employed, and 82% had a minimum of a college or university degree (Table 1).

**Table 1:** Demographic characteristics of study participants.

Participant characteristics	Pregnant women	Non-pregnant women
**Sample size**	426	426
**Age (years)**		
< 35	134 (78%)	134 (78%)
≥ 35	92 (22%)	92 (22%)
**Education**		
Primary and high school	74 (17%)	76 (18%)
College or university degree Post-Graduate degree or professional designation	276 (65%) 74 (17%)	283 (66%) 67 (16%)
**Marital status**		
Single	68 (16%)	154 (36%)
Married or common in low	336 (79%)	257 (60%)
Separated, divorced and widowed	16 (4%)	12 (3%)
**Employment status**		
Student	19 (4%)	38 (9%)
Employed	356 (84%)	314 (74%)
Retired or Unemployed	32 (8%)	44 (10%)
Other	16 (4%)	25 (6%)
**Income level**		
No income	1(0.2%)	4(1%)
Under $75,000	218 (51%)	231 (54%)
$75,000 to $109,999	110 (26%)	93 (22%)
Over $110,000	80 (19%)	68 (16%)
Prefer not to say	17 (4%)	30 (7%)
**Have children**	242(57%)	242 (57%)
**Born in Canada**	341 (80%)	356 (84%)

The sample of non-pregnant women was a subset of the general public sample. The characteristics of the general public sample were described in our previous published study [19]. Overall, 5,035 people answered the questionnaire. The internal consistency and completion time criteria were met by 4,601 of them. Out of 4,601 respondents, 81% were Canadian born, 54% were female, 58% were married or in a common-law relationship, and 58% had children. The majority of the sample’s participants were employed (63%), of European ancestry (63%), and under 50 years old (58%) (S5 Table). After matching, the baseline characteristics of the matched sample of non-pregnant women became similar to the pregnant women (Table 1).

### DCE preferences

After comparing different model specifications and testing the non-linear effects of attribute levels, in the final model, the false-negative rate, false-positive rate, and risk of miscarriage were effect coded, while out-of-pocket cost and time were treated as continuous. It was assumed that the coefficients of time and the levels of the false-negative rate, false-positive rate, and risk of miscarriage were random parameters that followed the normal distribution. The coefficient of the out-of-pocket cost was assumed to be non-random.

### Pregnant women - forced model

In the forced model, the utility coefficient for the time of the results was not statistically significant (S1 Table). Except for 0.5% risk of miscarriage and false-positive rate of 2%, the coefficients were statistically significant for all levels of the attributes. Preference weights for the levels for effect-coded attributes were in the order as expected and pregnant women preferred the lower levels of false negatives, false positives, and risk of miscarriage over the higher levels (S2 Fig and S1 Table).

### Pregnant women - unforced model

**Fig 1:**
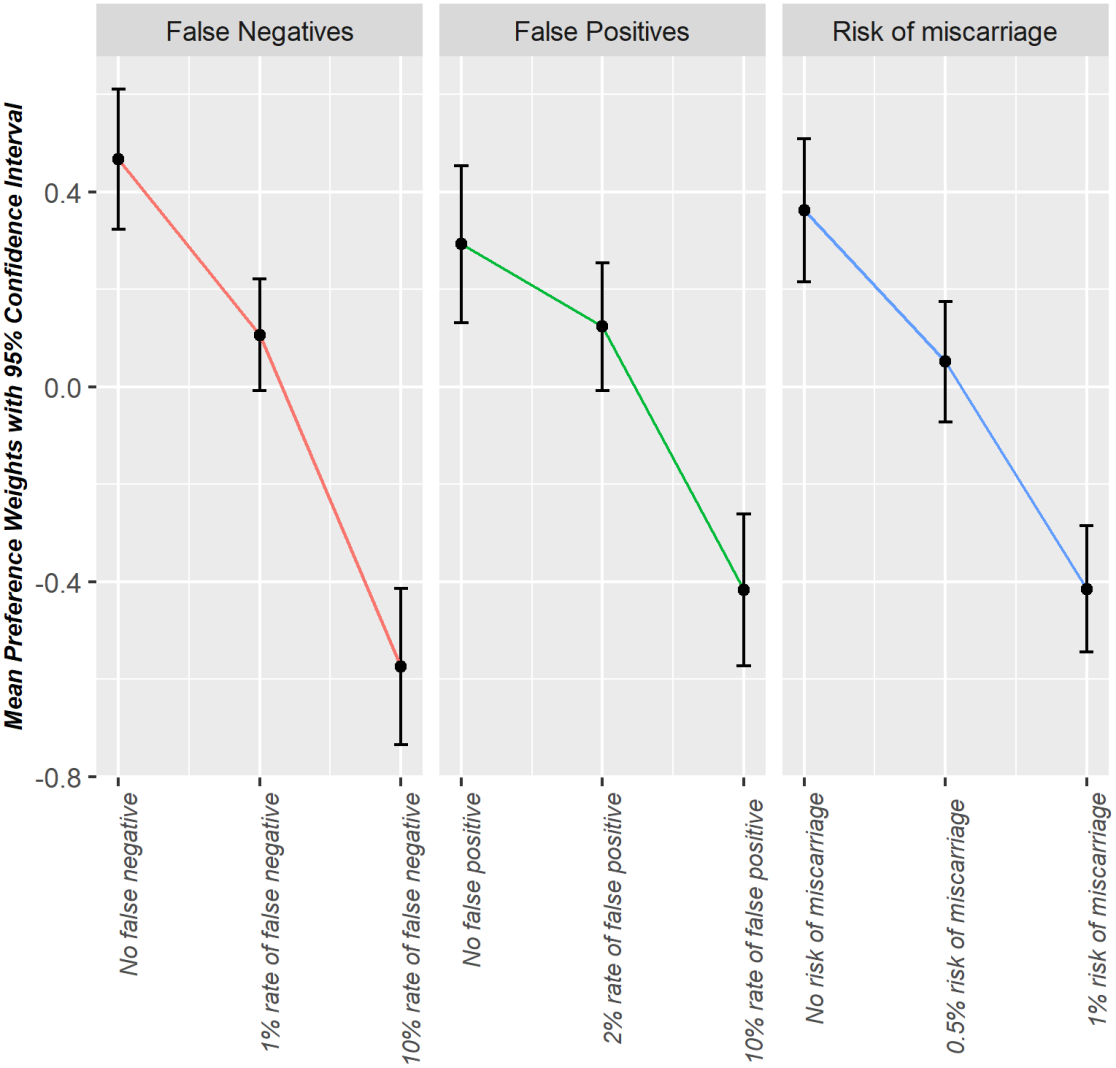
Preference weights with 95% confidence intervals; unforced model, pregnant women.

The results of the preference weight of the unforced model for the sample of pregnant women are presented in S2 Table and Fig 1. The coefficient for time of the results was also not significant in the unforced model. The utility coefficients were statistically significant for all levels of effect-coded attributes other than 0.5% risk of miscarriage. ASC parameter for screening was significant and positive, suggesting pregnant women’s systematic tendency for screening over no screening. The preference weights along with a 95% confidence interval, are also presented in Fig 1. Similar to the results from the forced model, pregnant women preferred the lower levels of false negatives, false positives, and risk of miscarriage over the higher levels.

Estimation results showed that pregnant women consider accuracy and safety when choosing between two screening strategies. We also evaluated the impact of the level of the risk on systematic preferences for screening by defining a constant specific to each level of the pre-screening risk. The results showed that pregnant women systematically preferred screening over no screening, and this systematic preference for screening became more pronounced as the level of risk increased.

### Preference heterogeneity

As shown in S1 Table and S2 Table, estimated variances for all random coefficients assigned to the attributes (both forced and unforced model) were statistically significant, indicating the existence of preference heterogeneity among the respondents. v were also significant differences in the distribution of estimated individual-specific parameters across the sample.

The false-negative rate was the most important attribute for pregnant women. There was no statistically significant difference between the relative importance of false-positive rate and risk of miscarriage, and thus both were the second most important attributes.

### Non-Pregnant women - unforced model

**Fig 2:**
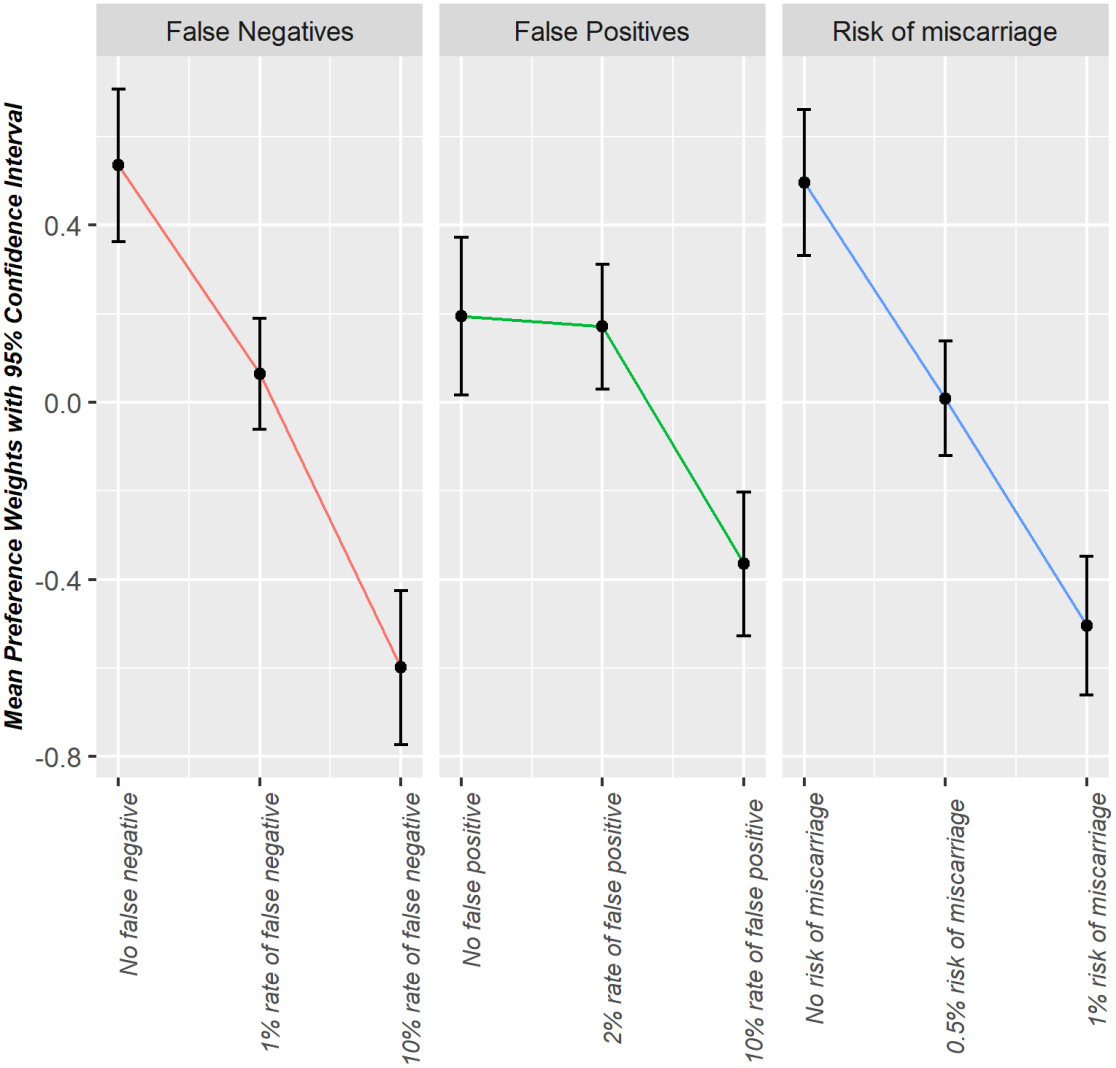
Preference weights with 95% confidence intervals; unforced model, matched sample of non-pregnant women.

The results of the unforced model for the matched sample of non-pregnant women are presented in S3 Table and Fig 2. Different from the pregnant women, the most important attribute for the non-pregnant women was false-negative rate and miscarriage rate. Similar to the pregnant women, time of the results was not significantly important. The preference weights for the levels of attributes were in the expected direction, and non-pregnant women preferred lower levels of false negatives, false positives, and risk of miscarriage over the higher levels. The impact of different levels of pre-screening risk on systematic preferences for screening was not statistically significant for non-pregnant women.

### Pooled model

In order to test the null hypothesis that preferences do not vary between the two populations of pregnant and non-pregnant women, we also ran a pooled model. A likelihood ratio (LR) test using the simulated log-likelihood value from the pooled model and the sum of the pregnant and non-pregnant models was used to determine whether there is a jointly significant difference in preferences. The results of the unforced model for the pooled sample are presented in S4 Table. The LR test (λ = 28) suggested a significant difference between the two samples’ preferences.

### Comparison between pregnant women and non-pregnant women samples

**Fig 3:**
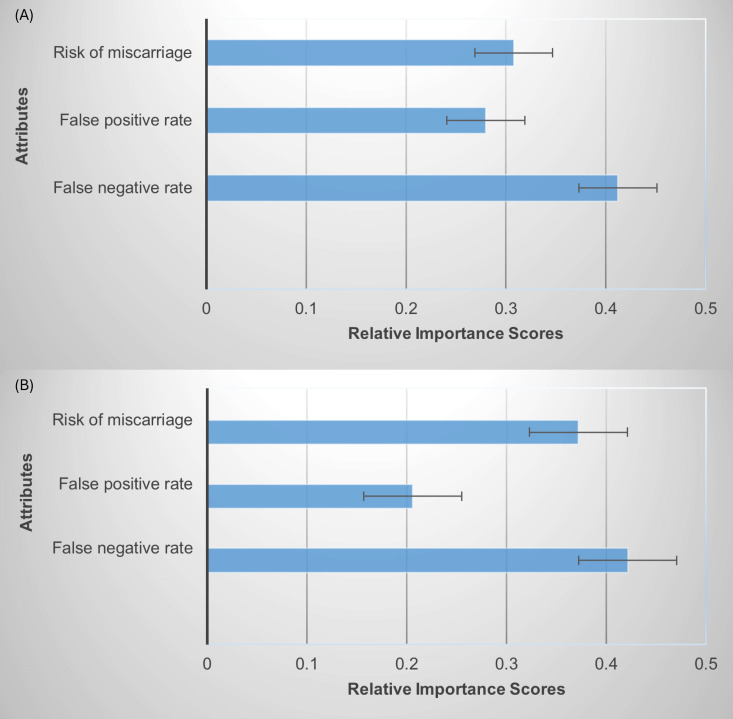
Relative importance scores of attributes across (A) the sample of pregnant women and (B) the sample of non-pregnant women.

ASC for choosing screening over opting out was positive and significant for both samples of pregnant women and non-pregnant women, indicating their systematic preferences for screening over no screening. Figs 3a and 3b show the relative importance scores of attributes for pregnant women and non-pregnant women based on the unforced model. S3 Fig shows the preference weights for both samples. Results showed that the false-negative rate was the most important attribute for pregnant women. For non-pregnant women, false-negative rate and risk of miscarriage were the most important attributes.

### Comparing willingness to pay between the two samples

The pregnant women’s and non-pregnant women’s WTP for improvement in attributes are presented in Table 2. As the results show, pregnant women were prepared to pay more than non-pregnant women for improvements in all three attributes. For instance, pregnant women were willing to pay $521 for reducing the number of missed babies with conditions from 100 to 0 out of every 1,000, while WTP for the same improvement in false-negative rate was $334 for non-pregnant women.

**Table 2:** Estimated monetary values of change in attributes for the matched samples of pregnant women and non-pregnant women.

	Pregnant women		Non-pregnant women	
	WTP ($CAD)	SE ($CAD)	WTP ($CAD)	SE ($CAD)
**False Negative Rate**				
Reduce the number of missed babies with conditions from 100 to 0 out of every 1,000	521	72	334	49
**False Positive Rate**				
Reduce the number of healthy babies with an inaccurate positive result from 100 to 0 out of 1,000	355	74	164	47
**Risk of Miscarriage**				
Reduce the risk of miscarriage from 10 to 0 out of 1,000 women	389	63	294	44
WTP willingness to pay; SE standard error; CAD Canadian dollars				

The additional money that respondents from both samples were willing to pay for the improved screening and diagnostic strategies (i.e., marginal WTP) compared to the “base-case” strategy was also calculated (Table 3). For the “base-case” strategy, the levels of false-negative rate, false-positive rate, and risk of procedure-related miscarriage were set at 10%, 10%, and 1%, respectively, and the results were available at 17 weeks in pregnancy. Improvement in all attributes resulted in an “optimal case” strategy in which the false positive rate, false-negative rate, and risk of procedure-related miscarriage were all set to zero, and time to results was at 11 weeks. In addition to the existing strategies, amniocentesis and CVS, we also calculated marginal WTP for NIPT, a relatively new highly sensitive screening test. False-negative and false-positive rates of 1% and 2% and time of the results at 11 weeks of pregnancy were assigned to NIPT.

**Table 3:** Marginal willingness to pay for different strategies for the matched samples of pregnant women and non-pregnant women.

Scenario	Attributes	Pregnant women MWTP (SE), CAD	Non-pregnant women MWTP (SE), CAD	Combinatorial test P value
Base case	10% false negative 10% false positive No risk of miscarriage 17 weeks time to results	Base	Base	
NIPT	1% false negative 2% false positive No risk of miscarriage 11 weeks time to results	695 (117)	424 (69)	0.02355
Amniocentesis	No false negative No false positive 0.5% risk of miscarriage 17 weeks time to results	720 (127)	355 (75)	0.00633
Chorionic villus sampling	No false negative No false positive 1% risk of miscarriage 14 weeks time to results	529 (123)	239 (72)	0.02098
Optimal case	No false negative No false positive No risk of miscarriage 11 weeks time to results	960 (129)	569 (82)	0.00509
MWTP marginal willingness to pay; CAD Canadian dollars; SE standard error; NIPT non-invasive prenatal test				

We employed the complete combinatorial test suggested by Poe et al. (2005) [27] to determine whether the marginal WTP difference between pregnant and non-pregnant women is statistically significant. The result of the Poe test is presented in Table 3, which suggested that the estimated WTP values are statistically different. As it is shown, pregnant women were willing to pay significantly more for better screening and diagnostic strategies compared to non-pregnant women. Pregnant women were prepared to pay an additional $695 for a strategy that reflects NIPT, while the estimated marginal WTP for non-pregnant women was $424. Marginal WTP for the “optimal case” was $960 for the sample of pregnant women and $569 for the matched sample of non-pregnant women.

### Comparing the uptake of different strategies between the two samples

We estimated the uptake of different strategies defined above for both samples. First, we set the levels of attributes for each strategy based on the available clinical information about the false positive and false negative rates, rate of miscarriage and time to results. Then using posterior draws for individual-level preferences, the utility score and the probability of choosing each strategy over no screening were calculated for each respondent in both samples. The likely uptake of each scenario was computed as a fraction of the respondents with an estimated probability of more than 50%. The uptake rate for all scenarios is presented in S4 Fig. In all scenarios, the uptake rate was slightly higher among the pregnant women sample.

**Fig 4:**
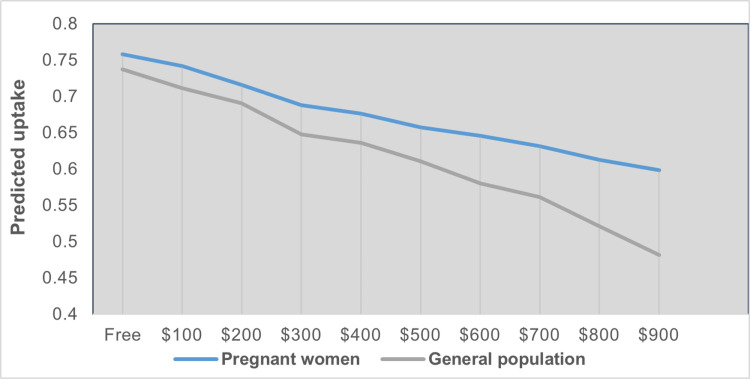
Impact of out-of-pocket cost on the predicted uptake of the “optimal case”.

We also evaluated the sensitivity of the predicted uptake rate to changes in the out-of-pocket cost from $0 to $900 for both samples. The results showed that the predicted uptake rate was more sensitive to changes in cost for the sample of non-pregnant women (Fig 4). When the out-of-pocket cost increased from 0 to $900, the estimated uptake rate of the “optimal case” for pregnant women decreased by 21%, while the decrease in the uptake rate for non-pregnant women was 35%.

### Measuring hypothetical bias

#### Pregnant women.

We converted the individual-level estimated probability of choosing different prenatal screening strategies over “no screening” to the choices using a 50% threshold as a binary classifier. In the province of British Columbia, for example, women have the option to undergo prenatal screening and diagnostic testing, such as the NIPT, amniocentesis, and CVS. Thus, we also asked pregnant women if they had or planned to have prenatal screening for chromosomal abnormalities in this pregnancy. The pregnant women’s responses to this question were compared to their predicted choices for prenatal screening strategies. After excluding the respondents who answered “Do not know” or “Prefer not to say,” out of 378 pregnant women, 105 women (28%) had a prenatal screening, and 185 women (49%) planned to have one in this pregnancy. The results of comparing the predicted uptake of NIPT, CVS, and amniocentesis with the pregnant women’s actual choice for prenatal screening are shown in Table 4. The main diagonal of Table 4 for each strategy represents the correspondence between the predicted choice in the hypothetical situation and their decision in the real world. Overall, the individual predicted choice of NIPT, CVS, and amniocentesis (versus no screening) agreed with the actual choice respectively for 74%, 73%, and 74% of the pregnant women sample. The consistency between the predicted and actual choices was more significant among respondents who had or planned to have prenatal screening. For the group of respondents who had screened, 80% (NIPT), 74% (CVS) and 75% (amniocentesis) were correctly predicted to choose a prenatal screening and for the women who planned to have prenatal screening, these percentages were 82% (NIPT), 77% (CVS), and 78% (amniocentesis).

**Table 4:** Comparing predicted and actual choices for prenatal screening; pregnant women and non-pregnant women.

	Pregnant women			Non-pregnant women	
**Predicted Choice**	***Have screened***	***Plan to screen***	***Not plan to screen***	***Have had screening***	***Have not had screening***
**Choosing NIPT**	84	151	45	78	62
**Not Choosing NIPT**	21	34	43	26	42
**True positive rate/True negative rate** ^**a**^	80%	82%	49%	75%	40%
**Choosing CVS**	78	142	31	73	57
**Not Choosing CVS**	27	43	57	31	47
**True positive rate/True negative rate**	74%	77%	65%	70%	45%
**Choosing amniocentesis**	79	145	34	77	58
**Not Choosing amniocentesis**	26	40	54	27	46
**True positive rate/True negative rate**	75%	78%	61%	74%	56%

^a^True positive rate = Rate of correctly predicted choices of women who had screening or plan to screen; True negative rate = Rate of correctly predicted choices of women without a screening plan

#### Non-Pregnant women.

The non-pregnant women were asked if they had been previously pregnant and, if they were, whether they had had a prenatal screening for chromosomal conditions in the previous pregnancy. We used their response to this question as a measure of their actual decisions. Similar to the pregnant women, the estimated probability of choosing different prenatal screening strategies (NIPT, CVS and amniocentesis) versus no screening was computed and then converted to the predicted choice for each individual non-pregnant woman. The results of comparing the predicted and actual choices for different strategies are presented in Table 4. Out of the 426 respondents in the non-pregnant women, 228 (53%) were previously pregnant. After excluding the respondents who answered “Don’t remember” or “Don’t know,” there were 208 women who specified their previous decision toward prenatal screening. Of this number, 104 (50%) had a previous screening, and 104 (50%) had decided not to have prenatal screening. As the results shown in Table 4, the predictive power of DCE estimates was lower for non-pregnant women. There was correspondence between the predicted choice of NIPT and actual decision for 75% of respondents who have had screening and 40% of the ones who have not had screening. These numbers were 70% and 45% for CVS and 74% and 56% for amniocentesis. Like pregnant women, the consistency between predicted and actual choices was higher among non-pregnant women who have undergone screening compared to those who reported no previous screening.

### Sensitivity analysis

We conducted a sensitivity analysis to assess the impact of excluding respondents who did not meet internal consistency criteria. The sensitivity analysis aimed to determine whether including these respondents significantly influenced the study’s outcomes. The results are presented in S6 Table. These results indicated that the inclusion of these respondents did not have a significant impact on the overall outcomes of the study. Specifically, the order, sign, statistical significance of attribute levels, and relative importance of attributes remained consistent after including data from this group of respondents.

## Discussion

Conducting the same DCE among pregnant women and members of the general public provided us with the unique opportunity to investigate the impact of being closer to real-life choice on preferences and WTP for prenatal screening and diagnostic testing. Some previous studies compared preferences for prenatal screening and diagnostic strategies between pregnant women and healthcare providers [28–30]. To our knowledge, no previous studies have compared the preferences of pregnant women with the general public.

Our findings showed that for pregnant women, the ability to detect chromosomal abnormalities was the most important attribute of prenatal screening strategies. False-positive rate and procedure-related risk of miscarriage were the second important characteristics. Some previous studies also have found that pregnant women put a great value on the accuracy of the screening and diagnostic strategies, especially the detection rate [31]. According to our analysis, time of results in terms of gestational age was not significant for pregnant women when they chose the prenatal screening and diagnostic strategy, which is consistent with the finding of some prior studies [32,33].

For the matched sample of non-pregnant women, false-negative rate and risk of miscarriage were the most important attributes. Same as pregnant women, time of results was not significant for non-pregnant women. Respondents from both samples placed a higher value on strategies with lower cost, higher detection rate, lower false positive rate, and lower risk of miscarriage, and revealed a systematic preference for screening over no screening. The results of our previous study showed that the false-negative rate was the most important attribute for the total sample of the general public. False-positive rate and procedure-related risk of miscarriage were also important for the members of the general population [19].

Comparing WTP for different screening and diagnostic strategies showed that pregnant women were willing to pay more for improvement in test characteristics than non-pregnant women and general public members [19]. The results of this study are in line with the assumptions of the CTL. Pregnant women, for whom all aspects of physiological distance to screening decisions were smaller, perceived risk differently from non-pregnant women and showed more willingness to pay for preventive treatment (prenatal screening). Previous studies have shown that smaller psychological distance is associated with an increase in perceived risks [13,34] and that people with higher perceived risk are willing to pay more for health interventions than those with lower perceived risk [35,36]. White et al. (2014) found that psychological proximity can impact threat perception. They showed that people perceived viruses that originated recently (psychologically close) as more dangerous than the ones discovered in distant years (psychologically distant) [13]. In addition, psychological proximity was associated with an increase in WTP for protections like a vaccine. In another study, Johnson (2018) [37] found that there was a link between a lower psychological distance and an increased motivation to engage in protective behaviours. According to Veldwijk et al. (2019), people’s choices for genetic screening for colorectal cancer vary depending on their psychological distance, particularly in terms of temporal and spatial distance [12]. Our results also suggested that non-pregnant women were more sensitive to increases in the cost of an optimal screening and diagnostic strategy compared to pregnant women. Based on our previous analysis, the sensitivity of demand for screening and diagnostic strategies, representing the degree of responsiveness to changes in cost, is also higher among the sample of the general public compared to pregnant women [19]. These findings are consistent with previous research, which indicated that, in general, individuals are more price-sensitive in hypothetical situations [38].

Another strength of our study is that we were able to measure and quantify hypothetical bias. We compared the predicted choices for different prenatal screening and diagnosis strategies with the real-world prior decisions of our pregnant and non-pregnant women samples. The results showed a more significant difference between the actual and predicted choice among non-pregnant women. Some previous studies have evaluated the external validity of DCEs in health economics [39], but only a few studies compared the comparing predictions to choices in the real world at an individual level [40–43]. The difference between the predicted and actual choices in both samples in our study was more significant than Mohammadi et al., with an overall agreement rate of 83% [40] and Lambooij et al. with 80% [41], and Salampessy et al. with 82% [42], and was closer to the results of Krucien et al. [43]. Similar to these previous studies [39], our study’s prediction based on DCE results was more sensitive than specific, indicating a higher predictive power of DCE for the respondents who chose to have prenatal screening. This suggested that prior screening experience or seeking information in preparation for the procedure may influence decision-making outcomes, with individuals who have undergone screening (or plan to) showing a greater alignment between their stated preferences and actual choices, potentially mitigating hypothetical bias.

This study showed that the difference in preferences between hypothetical and actual situations might be explained by the impact of psychological distance on the perceptions, preferences, and choices of respondents in stated preference studies. Implementing methods to decrease the perceived distance to the choice, such as using an educational tool, can help to mitigate or overcome the hypothetical bias in stated preference models [44].

One potential limitation of this study was that both questionnaires were administrated online, and pregnant women and members of the general public without access to the internet were excluded. As such, these samples may have been prone to selection biases. Also, similar to other DCEs, the results depend on a limited number of attributes that could be included in the study. As such, there might be some other considerations in individuals’ real-life decision-making for prenatal testing that is missing from our study. However, by following the recommendations for good practice in designing DCEs and employing “think-aloud” interviews as a pre-testing method and two rounds of pilot studies, we tried to minimize this issue. Next, in terms of generalizability, our study focused on prenatal screening and diagnosis. This raises the question of whether our findings generalize to other areas. Considering that in many areas of healthcare, accounting for the preferences of different stakeholders is significant for informed policy decision-making, it may be of interest to examine the impact of psychological distance on stated preferences in other areas. Additionally, there was a slight difference in wording between surveys for pregnant women and the general public, from which the sample of non-pregnant individuals was selected. In the general public survey, which included male respondents, the wording was, “Please imagine you or your family members are pregnant and considering prenatal screening and diagnosis,” but in the survey for pregnant women it was “Please imagine that you are considering prenatal screening and diagnosis.” Acknowledging the difference, we believe that after matching the two samples by demographic characteristics, it is reasonable to assume that non-pregnant female respondents would empathize with the perspective of a pregnant women and project themselves into that role when responding to the task. In fact, we chose the method of matching the two samples, to ensure that the perspectives elicited were as close as possible. Furthermore, we matched the two groups, considering factors like having children. This approach aimed to avoid confounding effects related to not being pregnant and not having children simultaneously. Although preferences may vary between non-pregnant women without prior pregnancies and pregnant women, we could not identify the specific factors contributing to these distinctions if we didn’t match the two samples. Our study primarily focused on assessing the influence of psychological distance, particularly temporal distance. Even though some non-pregnant women had children, their current non-pregnant status can lead to distinct preferences for prenatal screening due to the temporal gap, affecting hypothetical bias. In essence, the passage of time creates a unique psychological context, which we aimed to investigate. Our prior research among the general public indicated that having children was associated with a reduced likelihood of choosing screening over no screening [19]. Future studies can delve deeper into the impact of having or not having children on prenatal screening preferences and hypothetical bias.

## Conclusion

According to the findings of this study, although both pregnant women and non-pregnant women placed a higher value on strategies with higher detection rate, lower false-positive rate, lower risk of miscarriage, and lower cost, pregnant women were willing to pay more for improvement in these strategy characteristics and less sensitive to changes of strategy cost. We also evaluated the difference in the predictive power of DCE (hypothetical bias) between the two samples. This difference can be explained by their psychological distance from the decision. The identified hypothetical bias emphasized the special attention paid to the perceived psychological distance in designing stated preference studies. Furthermore, our findings of Canadian pregnant women’s preferences for different aspects of prenatal screening and diagnosis have important clinical and policy implications for the development of more patient-centered approaches to prenatal care. These results can be used in designing and implementing prenatal screening and diagnostic strategies in a way that more meet the needs of the target groups. Additionally, they can facilitate predicting the acceptability and uptake of emerging technologies in prenatal screening and diagnosis for chromosomal abnormalities, thereby contributing to more informed decision-making processes in healthcare delivery and policy formulation.

## Supporting information

S1 Fig A sample of DCE choice sets (forced and unforced). (DOCX)

S2 Fig Preference weights with 95% confidence intervals; pregnant women-forced model. (DOCX)

S3 Fig Preference weights from unforced model; pregnant and non-pregnant women. (DOCX)

S4 Fig Predicted uptake for different strategies; pregnant women and non-pregnant women samples. (DOCX)

S1 Table Results of the forced model; pregnant women. (DOCX)

S2 Table Results of the unforced model; pregnant women. (DOCX)

S3 Table Results of the unforced model; matched sample of non-pregnant women. (DOCX)

S4 Table Results of the pooled model. (DOCX)

S5 Table General public sample demographic characteristics. (DOCX)

S6 Table Results of the sensitivity analysis; forced model including pregnant women respondents who failed the internal consistency check. (DOCX)

S1 File Full survey questionnaire. (DOCX)

S2 File Sample size calculation formula. (DOCX)

S3 File Utility function and mWTP calculation. (DOCX)
