# Supplementary figures and images for: The impact of psychological distance on preferences for prenatal screening and diagnosis for chromosomal abnormalities: A hierarchical Bayes analysis of a discrete choice experiment
Source: PLoS One. 2025 May 23;20(5):e0324370. doi: 10.1371/journal.pone.0324370 (PMC12101744; doi:10.1371/journal.pone.0324370)

**
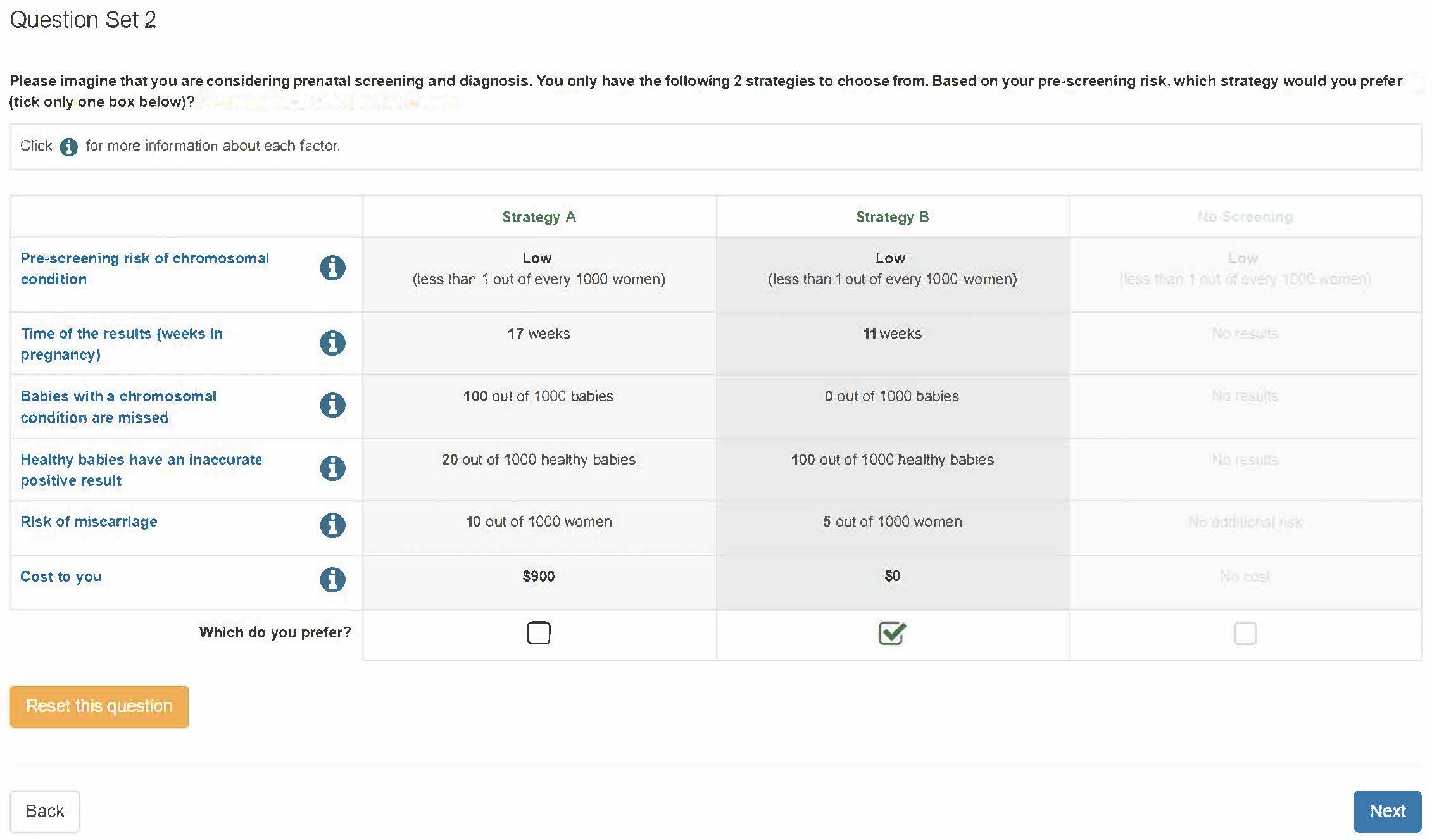
**

**
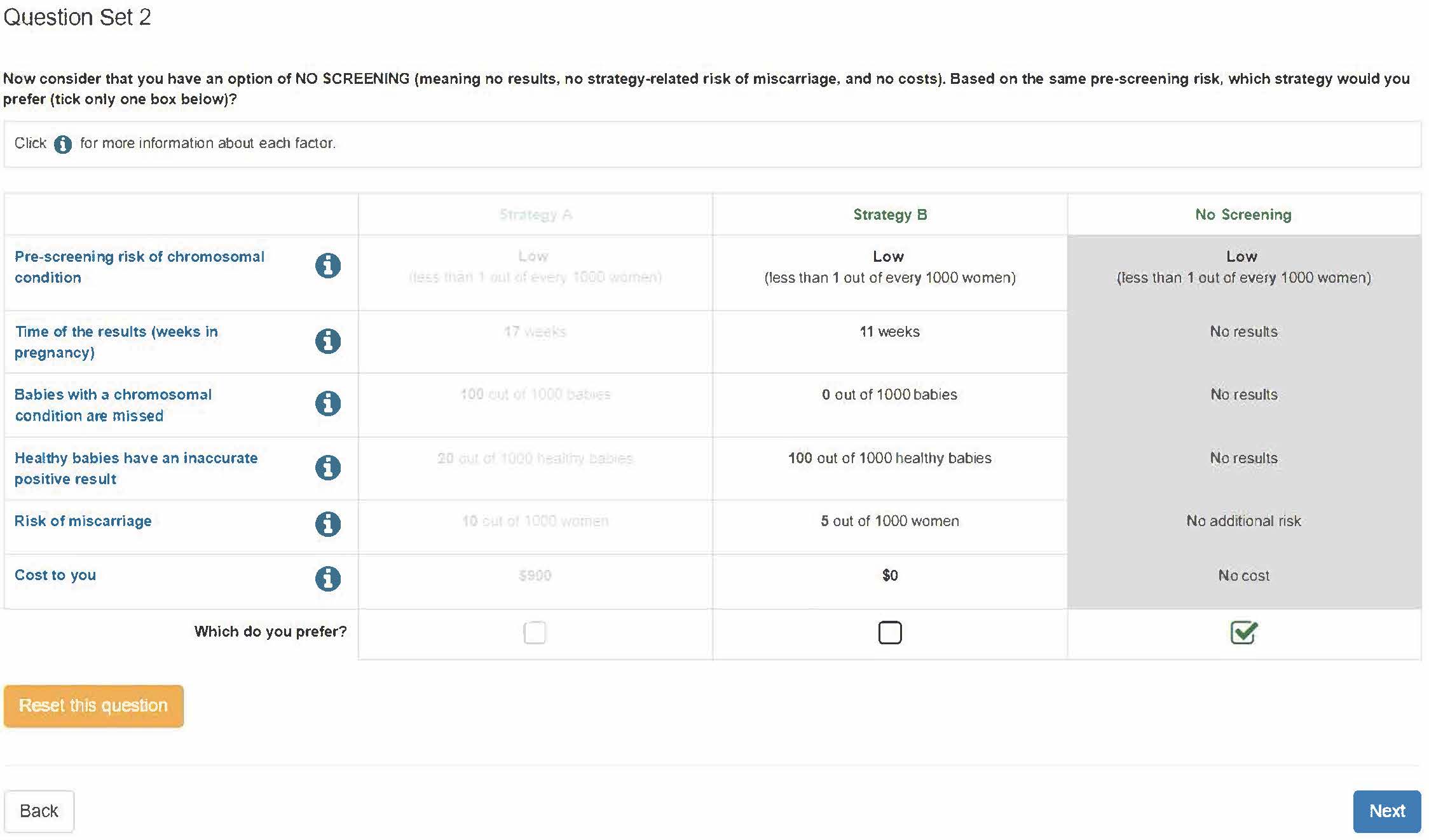
**

**S1 FIG.** **A sample of DCE choice sets (forced and unforced).**

Supplement: S1 Fig — (DOCX) [file pone.0324370.s001.docx]

**
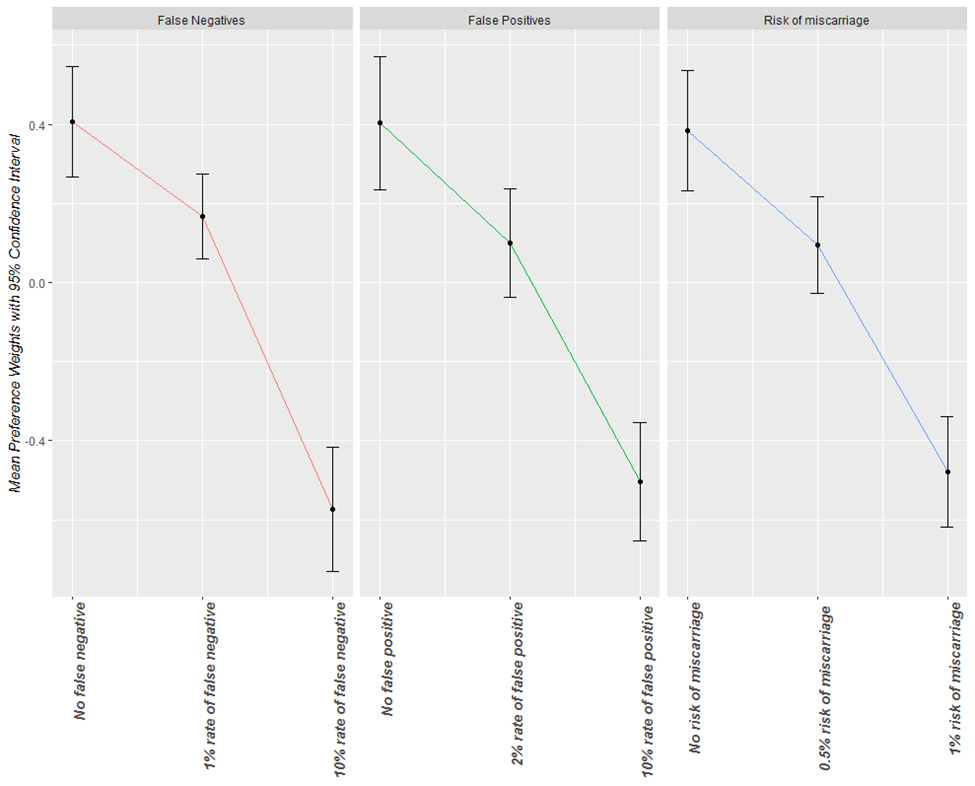
**

**S2 Fig. Preference weights with 95% confidence intervals; pregnant women-forced model.**

Supplement: S2 Fig — (DOCX) [file pone.0324370.s002.docx]
