# Supplementary material for: The impact of psychological distance on preferences for prenatal screening and diagnosis for chromosomal abnormalities: A hierarchical Bayes analysis of a discrete choice experiment
Source: PLoS One. 2025 May 23;20(5):e0324370. doi: 10.1371/journal.pone.0324370 (PMC12101744; doi:10.1371/journal.pone.0324370)
Supplement: S4 Fig — (DOCX) [file pone.0324370.s004.docx]

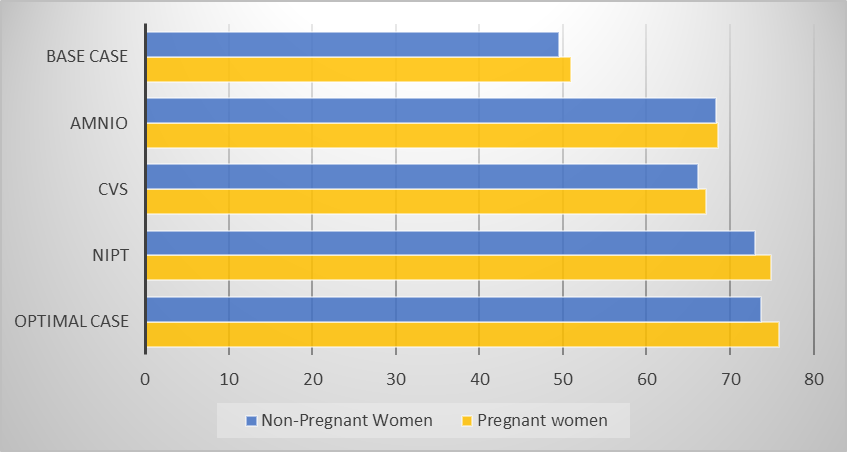


**S4 Fig. Predicted uptake for different strategies; pregnant women and non-pregnant women samples.**
