# Supplementary material for: The impact of psychological distance on preferences for prenatal screening and diagnosis for chromosomal abnormalities: A hierarchical Bayes analysis of a discrete choice experiment
Source: PLoS One. 2025 May 23;20(5):e0324370. doi: 10.1371/journal.pone.0324370 (PMC12101744; doi:10.1371/journal.pone.0324370)
Supplement: S1 Table — (DOCX) [file pone.0324370.s005.docx]

**S1 Table. Results of the forced model; pregnant women.**

| **Attributes** | **Mean of posterior/ coefficient** | **SE** | **Variance of posterior** | **SE** |
| --- | --- | --- | --- | --- |
| ***Random variables*** | | | | |
| Babies with a chromosomal condition are missed |  |  |  |  |
| 0 out of every 1000 | 0.4060 | 0.0713 | 1.0501 | 0.4893 |
| 10 out of every 1000 | 0.1667 | 0.0549 | 0.3341 | 0.0632 |
| 100 out of every 1000 | -0.5727 | 0.0801 | 1.3898 | 0.2118 |
| Healthy babies have an inaccurate positive result |  |  |  |  |
| 0 out of every 1000 | 0.4034 | 0.0861 | 1.8912 | 0.4977 |
| 20 out of every 1000 | 0.1001 | 0.0696 | 0.8181 | 0.1499 |
| 100 out of every 1000 | -0.5036 | 0.0762 | 1.0586 | 0.1783 |
| Risk of miscarriage |  |  |  |  |
| 0 out every 1000 | 0.3839 | 0.0779 | 1.0813 | 0.4151 |
| 5 out of every 1000 | 0.0946 | 0.0622 | 0.4361 | 0.0854 |
| 10 out of every 1000 | -0.4785 | 0.0712 | 0.7166 | 0.1372 |
| Time to results (weeks) | 0.0079 | 0.0211 | 0.093 | 0.0111 |
| ***Fixed variables*** | | | | |
| Cost to you | -0.0023 | 0.0001 |  |  |
| Simulated log-likelihood value: -2,272  SE Standard error | | | | |
