# Supplementary material for: The impact of psychological distance on preferences for prenatal screening and diagnosis for chromosomal abnormalities: A hierarchical Bayes analysis of a discrete choice experiment
Source: PLoS One. 2025 May 23;20(5):e0324370. doi: 10.1371/journal.pone.0324370 (PMC12101744; doi:10.1371/journal.pone.0324370)
Supplement: S2 Table — (DOCX) [file pone.0324370.s006.docx]

**S2 Table. Results of the unforced model; pregnant women.**

| **Attributes** | **Mean of posterior/ coefficient** | **SE** | **Variance of posterior** | **SE** |
| --- | --- | --- | --- | --- |
| ***Random variables*** | | | | |
| Alternative-specific constant | 1.953 | 0.289 | 10.070 | 2.647 |
| Babies with a chromosomal condition are missed |  |  |  |  |
| 0 out of every 1000 | 0.467 | 0.074 | 0.926 | 0.525 |
| 10 out of every 1000 | 0.107 | 0.058 | 0.401 | 0.073 |
| 100 out of every 1000 | -0.574 | 0.082 | 1.326 | 0.198 |
| Healthy babies have an inaccurate positive result |  |  |  |  |
| 0 out of every 1000 | 0.293 | 0.082 | 1.448 | 0.525 |
| 20 out of every 1000 | 0.124 | 0.067 | 0.614 | 0.110 |
| 100 out of every 1000 | -0.417 | 0.080 | 1.142 | 0.181 |
| Risk of miscarriage |  |  |  |  |
| 0 out every 1000 | 0.363 | 0.075 | 0.941 | 0.378 |
| 5 out of every 1000 | 0.052 | 0.063 | 0.441 | 0.083 |
| 10 out of every 1000 | -0.415 | 0.066 | 0.490 | 0.092 |
| Time to results (weeks) | -0.028 | 0.021 | 0.076 | 0.008 |
| ***Fixed variables*** | | | | |
| Cost to you | -0.002 | 0.0001 | _ | _ |
| Simulated log-likelihood value: -3,305  SE, Standard error |  |  |  |  |
