# Supplementary material for: The impact of psychological distance on preferences for prenatal screening and diagnosis for chromosomal abnormalities: A hierarchical Bayes analysis of a discrete choice experiment
Source: PLoS One. 2025 May 23;20(5):e0324370. doi: 10.1371/journal.pone.0324370 (PMC12101744; doi:10.1371/journal.pone.0324370)
Supplement: S3 Table — (DOCX) [file pone.0324370.s007.docx]

**S3 Table. Results of the unforced model; matched sample of non-pregnant women.**

| **Attributes** | **Mean of posterior/ coefficient** | **SE** | **Variance of posterior** | **SE** |
| --- | --- | --- | --- | --- |
| ***Random variables*** | | | | |
| Alternative-specific constant | 2.013 | 0.367 | 24.467 | 4.562 |
| Babies with a chromosomal condition are missed |  |  |  |  |
| 0 out of every 1000 | 0.535 | 0.088 | 1.358 | 0.481 |
| 10 out of every 1000 | 0.063 | 0.064 | 0.323 | 0.063 |
| 100 out of every 1000 | -0.599 | 0.089 | 1.285 | 0.206 |
| Healthy babies have an inaccurate positive result |  |  |  |  |
| 0 out of every 1000 | 0.194 | 0.091 | 1.625 | 0.472 |
| 20 out of every 1000 | 0.171 | 0.072 | 0.563 | 0.108 |
| 100 out of every 1000 | -0.365 | 0.083 | 1.011 | 0.176 |
| Risk of miscarriage |  |  |  |  |
| 0 out every 1000 | 0.496 | 0.084 | 0.981 | 0.397 |
| 5 out of every 1000 | 0.009 | 0.066 | 0.368 | 0.075 |
| 10 out of every 1000 | -0.505 | 0.080 | 0.670 | 0.127 |
| Time to results (weeks) | -0.040 | 0.024 | 0.101 | 0.013 |
| ***Fixed variables*** | | | | |
| Cost to you | -0.003 | 0.0002 | _ | _ |
| Simulated log-likelihood value: -3,030  SE, Standard error |  |  |  |  |
