# Supplementary material for: The impact of psychological distance on preferences for prenatal screening and diagnosis for chromosomal abnormalities: A hierarchical Bayes analysis of a discrete choice experiment
Source: PLoS One. 2025 May 23;20(5):e0324370. doi: 10.1371/journal.pone.0324370 (PMC12101744; doi:10.1371/journal.pone.0324370)
Supplement: S4 Table — (DOCX) [file pone.0324370.s008.docx]

**S4 Table. Results of the pooled model.**

| **Attributes** | **Mean of posterior/ coefficient** | **SE** | **Variance of posterior** | **SE** |
| --- | --- | --- | --- | --- |
| ***Random variables*** | | | | |
| Alternative-specific constant | -1.758 | 0.216 | 14.822 | 2.231 |
| Babies with a chromosomal condition are missed |  |  |  |  |
| 0 out of every 1000 | 0.460 | 0.051 | 0.871 | 0.359 |
| 10 out of every 1000 | 0.074 | 0.039 | 0.237 | 0.036 |
| 100 out of every 1000 | -0.534 | 0.056 | 1.065 | 0.120 |
| Healthy babies have an inaccurate positive result |  |  |  |  |
| 0 out of every 1000 | 0.214 | 0.055 | 1.187 | 0.357 |
| 20 out of every 1000 | 0.131 | 0.045 | 0.459 | 0.065 |
| 100 out of every 1000 | -0.345 | 0.051 | 0.820 | 0.101 |
| Risk of miscarriage |  |  |  |  |
| 0 out of every 1000 | 0.400 | 0.050 | 0.727 | 0.277 |
| 5 out of every 1000 | 0.013 | 0.042 | 0.277 | 0.044 |
| 10 out of every 1000 | -0.414 | 0.049 | 0.424 | 0.064 |
| Time to results (weeks) | -0.024 | 0.014 | 0.593 | 0.006 |
| ***Fixed variables*** | | | | |
| Cost to you | -0.0024 | 0.0001 | _ | _ |
| Simulated log-likelihood value: -6320.5  SE, Standard error |  |  |  |  |
