# Supplementary material for: The impact of psychological distance on preferences for prenatal screening and diagnosis for chromosomal abnormalities: A hierarchical Bayes analysis of a discrete choice experiment
Source: PLoS One. 2025 May 23;20(5):e0324370. doi: 10.1371/journal.pone.0324370 (PMC12101744; doi:10.1371/journal.pone.0324370)
Supplement: S5 Table — (DOCX) [file pone.0324370.s009.docx]

### **S5 Table. General public sample demographic characteristics, n=4601.**

| Participant characteristics | N (%) |
| --- | --- |
| Female | 2492 (54.0) |
| Age (years) |  |
| 18–24 | 430 (9.0) |
| 25-29 | 400 (9.0) |
| 30–34 | 496 (11.0) |
| 35–39 | 489 (11.0) |
| 40–49 | 837 (18.0) |
| 50–59 | 939 (20.0) |
| 60+ | 969 (21.0) |
| Born in Canada | 3718 (81.0) |
| Education (completed level) |  |
| Primary school | 42 (1.0) |
| High school | 1008 (22.0) |
| College or technical/trade | 1498 (33.0) |
| University | 1345 (29.0) |
| Post-graduate or professional designation | 650 (14.0) |
| Ethnicity |  |
| Aboriginal | 213 (5.0) |
| African | 76 (2.0) |
| East Asian | 413 (9.0) |
| European | 2913 (63.0) |
| Latin American | 95 (2.0) |
| South Asian | 150 (3.0) |
| West Asian | 53 (3.0) |
| Other | 721 (16.0) |
| Marital status |  |
| Single | 1312 (28.5) |
| Married | 2234 (48.5) |
| Common law | 466 (10.0) |
| Separated | 113 (2.0) |
| Divorced | 302 (6.5) |
| Widowed | 133 (3.0) |
| Employment Status |  |
| Student | 266 (6.0) |
| Employed (full-time) | 2283 (50.0) |
| Employed (part-time) | 594 (13.0) |
| Retired | 811 (18.0) |
| Unemployed | 358 (8.0) |
| Other | 231 (5.0) |
| Income level |  |
| No income | 57 (1.0) |
| Under $35,000 | 836 (18.0) |
| $35,000 to $74,999 | 1475 (32.0) |
| $75,000 to $109,999 | 997 (22.0) |
| Over $110,000 | 842 (18.0) |
| Have children | 2667 (58.0) |
