# Supplementary material for: The impact of psychological distance on preferences for prenatal screening and diagnosis for chromosomal abnormalities: A hierarchical Bayes analysis of a discrete choice experiment
Source: PLoS One. 2025 May 23;20(5):e0324370. doi: 10.1371/journal.pone.0324370 (PMC12101744; doi:10.1371/journal.pone.0324370)
Supplement: S6 Table — (DOCX) [file pone.0324370.s010.docx]

**S6 Table.** **Results of the sensitivity analysis; forced model including pregnant women respondents who failed the internal consistency check.**

| **Attributes** | **Mean of posterior/ coefficient** | **SE** | **P value** | **Variance of posterior** | **SE** | **P Value** |
| --- | --- | --- | --- | --- | --- | --- |
| ***Random variables*** | | | | | | |
| Babies with a chromosomal condition are missed |  |  |  |  |  |  |
| 0 out of every 1000 | 0.3055 | 0.0558 | <0.001 | 0.7486 | 0.3894 | 0.0546 |
| 10 out of every 1000 | 0.1305 | 0.0454 | 0.004 | 0.2632 | 0.0458 | <0.001 |
| 100 out of every 1000 | -0.4360 | 0.0619 | <0.001 | 0.9902 | 0.1379 | <0.001 |
| Healthy babies have an inaccurate positive result |  |  |  |  |  |  |
| 0 out of every 1000 | 0.3200 | 0.0675 | <0.001 | 1.3261 | 0.3265 | <0.001 |
| 20 out of every 1000 | 0.0166 | 0.0552 | 0.7636 | 0.6313 | 0.1039 | <0.001 |
| 100 out of every 1000 | -0.3366 | 0.0574 | <0.001 | 0.6845 | 0.1073 | <0.001 |
| Risk of miscarriage |  |  |  |  |  |  |
| 0 out every 1000 | 0.2351 | 0.0616 | <0.001 | 0.8079 | 0.3224 | 0.0122 |
| 5 out of every 1000 | 0.0983 | 0.0488 | 0.04399 | 0.2913 | 0.0533 | <0.001 |
| 10 out of every 1000 | -0.3334 | 0.0554 | <0.001 | 0.5046 | 0.0888 | <0.001 |
| Time to results (weeks) | 0.0035 | 0.0175 | 0.8415 | 0.0754 | 0.0080 | <0.001 |
| ***Fixed variables*** | | | | | | |
| Cost to you | -0.0015 | 0.0001 | <0.001 |  |  |  |
| Simulated log-likelihood value: -2,9051 | | | | | | |
| SE Standard error | | | | | | |
|  |  |  |  |  |  |  |
