# Supplementary material for: The impact of psychological distance on preferences for prenatal screening and diagnosis for chromosomal abnormalities: A hierarchical Bayes analysis of a discrete choice experiment
Source: PLoS One. 2025 May 23;20(5):e0324370. doi: 10.1371/journal.pone.0324370 (PMC12101744; doi:10.1371/journal.pone.0324370)
Supplement: S1 File — (DOCX) [file pone.0324370.s011.docx]

**SURVEY INTRODUCTION**

**Welcome!**

Thank you for your interest in our study. The goal of this survey is to find out what is important to you when deciding on prenatal screening and diagnostic strategies.This information will help improve these strategies in the future. Before you agree to participate in this survey, please read the following information.

**SURVEY INFORMATION**

**Who is conducting the study?**

Researchers at the University of British Columbia (UBC) and the Centre for Health Evaluation and Outcome Sciences (CHÉOS) are conducting this study. This study has obtained ethical approval from UBC (H17-00591).

**Who is funding this study?**

The study is funded by St. Paul’s Hospital Foundation, CHÉOS, and BGI (a global genomics organization).

**Why are we doing this study?**

The purpose of this project is to find out which aspects of prenatal screening and diagnostic strategies are most important to pregnant women and members of the general public. This information will help policy makers understand how prenatal screening and diagnostic strategies can be improved.

**How is the study done?**

Participating in this study will involve taking an online survey which is expected to take **20 minutes** to complete. You will be asked to consider your decision with regards to prenantal screening and diagnosis. The survey will ask you to pick between two options.

**Will my participation be kept confidential?**

Your rights to privacy are protected. No information that discloses your identity will be released or published without your consent unless required by law. All documents will be identified only by code number. Anonymized data will be stored on a network accessible through password protected computers.

**What are the risks of participating?**

We do not think you face any risks by participating in this study. Participating in this study will not affect your ability to access any health care services including prenatal screening or diagnosis in the future.

**What are the benefits of participating?**

You may learn more about prenatal screening and diagnosis. In the future, others may benefit from what we learn in this study.

**What will happen with the results of the study?**

The results of the study will be published in journal articles, and presented at conferences. No one will be able to identify you in any reports or publications. If you would like a copy of the results once the study has finished, please contact [preferences@cheos.ubc.ca](mailto:preferences@cheos.ubc.ca).

**Who can I contact if I have a question or concern about the study?**

If you have any questions about the study, please contact the Principal Investigator or Project Manager at [preferences@cheos.ubc.ca](mailto:preferences@cheos.ubc.ca).

If you have any concerns or complaints about your rights as a research participant and/or your experiences while participating in this study, contact the Research Participant Complaint Line in the UBC Office of Research Ethics at 604-822-8598 or if long distance e-mail RSIL@ors.ubc.ca or call toll free 1-877-822-8598.

If you have any questions or concerns about prenatal screening and diagnosis, please click [here](http://www.perinatalservicesbc.ca/).

**Participant Consent!**

Taking part in this study is entirely up to you. You have the right to refuse to participate in this study. If you decide to take part, you may choose to pull out of the study at any time without giving a reason and without any penalty.

If you understand what is involved and you wish to participate in this study, please click “Next” to continue. By clicking “Next” and doing the survey, it will be assumed that consent is given. It is recommended that you print and/or save a copy of this consent form.

**Survey Overview**

This survey has 3 sections:

**Section 1 - Introduction:**

- Background: Chromosomal conditions and prenatal screening and diagnostic strategies
- Factor Description: Characteristics of prenatal screening and diagnostic strategies

**Section 2 – Questionnaire:** 11 question sets asking you to choose between 2 strategies

**Section 3 - About Me:** 17 questions about you and your experiences with pregnancy

There are no right or wrong answers. We are only interested in your opinion.

**BACKGROUND**

This page gives you information about (I) pregnancy timelines, (II) chromosomal conditions, (III) prenatal screening and diagnostic strategies, and (IV) decisions following screening and diagnostic results. This information will help you complete the survey.

**I. Pregnancy timelines**

A typical pregnancy last about 40 weeks or 9 months. The earliest a screening test can be done is at 9 weeks of pregnancy.

**
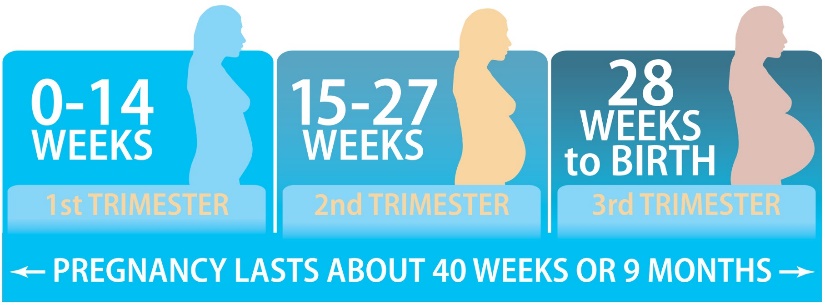
**

(Image adapted from [*http://www.perinatalservicesbc.ca/Documents/Resources/HealthPromotion/PregnancyPassport/PregnancyPassport.pdf*](http://www.perinatalservicesbc.ca/Documents/Resources/HealthPromotion/PregnancyPassport/PregnancyPassport.pdf))

**II. What are chromosomal conditions?**

Chromosomes are present in almost all human cells and store our genetic information. Extra copies of chromosomes can occasionally be present. The effects of this can sometimes result in chromosomal conditions such as:

| **Name** | **Cause** | **Characteristics** |
| --- | --- | --- |
| Down Syndrome (DS) | Extra copy of chromosome 21 | Results in intellectual disabilities and developmental delays. Some health problems such as vision and hearing problems, and heart, stomach and bowel defects may arise. People with DS can lead fulfilling lives and usually live into their 50’s. |
| Edward’s Syndrome | Extra copy of chromosome 18 | Babies rarely live more than a few days or months because of serious heart and brain defects and poor growth before and after birth. |
| Other rare conditions | Extra copy or missing copy of chromosome; duplicated or missing sections of chromosome | Results in intellectual disabilities, developmental delays, or other health problems. |

**III. What are prenatal screening and diagnostic strategies?**

Prenatal screening and diagnosis is not mandatory but is offered as a choice to all pregnant women in Canada. Prenatal screening and diagnostic strategies can consist of screening tests and/or diagnostic tests depending on the characteristics of the woman, such as:

- Age
- Health
- Family history
- How far along she is in the pregnancy
- Whether she is carrying more than one baby
- Personal choice

| **Type of test** | **What do they tell?** | **How are they done?** | **What do test results mean?** |
| --- | --- | --- | --- |
| Screening tests | Identify women who have an increased chance of having a baby with a chromosomal condition. These tests do not give a yes/no answer. | Blood tests and/or an ultrasound | **Positive (abnormal) result:** Increased chance of having a chromosomal condition; can confirm with diagnostic test  **Negative (normal) result:** NO increased chance of a chromosomal condition |
| Diagnostic tests | Will confirm whether or not the baby has a chromosomal condition. These tests give a yes/no answer. | Invasive test such as amniocentesis (needle is passed through the woman’s abdomen to obtain the sample). These tests carry a small risk of pregnancy loss (miscarriage). | **Positive (abnormal) result:** Chromosomal condition is confirmed  **Negative (normal) result:** Chromosomal condition is NOT found |
|  |  |  |  |

**IV. Decisions following screening and diagnostic results**

One possibility following a positive (abnormal) screening result is the option to take a diagnostic test. If the diagnostic test confirms a positive result for a chromosomal condition, a decision to end the pregnancy (abortion) is possible. Abortions are rarely done after **24 weeks** of pregnancy. Risks are lower when an abortion is done early in pregnancy. For more information, please click [here](https://www.healthlinkbc.ca/health-topics/tw1040).

**Factor Description**

The following are 6 important factors that may affect your decision on prenatal screening and diagnostic strategies. You will be asked to consider these factors in the survey to come.

- **Pre-screening risk of chromosomal condition:** Before screening, depending on characteristics such as age, personal or family history, single or twin pregnancy, etc., you may be told that your chance of having a baby with a chromosomal condition is:

1. **Low** (less than 1 out of every 1000 women)
2. **Intermediate** (between 1 out of every 1000 and 10 out of every 1000 women)
3. **High** (higher than 10 out of every 1000 women)

- **Time of the results (weeks in pregnancy):** For different strategies, you could get results at:

1. **11 weeks** in pregnancy
2. **14 weeks** in pregnancy
3. **17 weeks** in pregnancy

(*A typical pregnancy lasts about 40 weeks. Abortions are rarely done after 24 weeks of pregnancy. Risks are lower when an abortion is done early in pregnancy.)

- **Babies with a chromosomal condition are missed:** Sometimes results come back normal, but there is still a chance that a baby with a chromosomal condition is missed. For different strategies, the chances are:

1. **0 out of every 1000** babies with a condition are missed
2. **10 out of every 1000** babies with a condition are missed
3. **100 out of every 1000** babies with a condition are missed

- **Healthy babies have an inaccurate positive result:** Sometimes results come back abnormal, but there is still a chance that the baby does not have a chromosomal condition. For different strategies, the chances are:

1. **0 out of every 1000** healthy babies have an inaccurate positive result
2. **20 out of every 1000** healthy babies have an inaccurate positive result
3. **100 out of every 1000** healthy babies have an inaccurate positive result

- **Risk of miscarriage:** Some strategies can cause miscarriage. For different strategies, the chances of miscarriage are:

1. **0 out every 1000** women
2. **5 out of every 1000** women
3. **10 out of every 1000** women

- **Cost to you:** Some strategies may be offered free of charge, while others may require out-of-pocket payment. The range of costs that you may be charged include:

1. **$0**
2. **$300**
3. **$900**

# QUESTIONNAIRE

# SECTION A: Questionnaire

This section has 11 question sets. Each question set will ask you to choose between 2 prenatal screening and diagnostic strategies. These strategies are hypothetical. There are no right or wrong answers to the questions.

**Question 1 of 11**

a) Please imagine that you are considering prenatal screening and diagnosis. **You only have the following 2 strategies to choose from. Based on your pre-screening risk, which strategy would you prefer (tick only one box below)?**

|  | **Strategy A** | **Strategy B** |
| --- | --- | --- |
| Your pre-screening risk of chromosomal condition | **High**  *(Higher than 10 out of 1000 women)* | **High**  *(Higher than 10 out of 1000 women)* |
| Time of results (weeks in pregnancy) | **14** weeks | **11** weeks |
| Babies with an condition are missed (false negative) | **10** out of 1000 babies | **0** out of 1000 babies |
| Healthy babies have an inaccurate positive result (false positive) | **20** out of 1000 healthy babies | **0** out of 1000 healthy babies |
| Risk of miscarriage | **10** out of 1000 women | **0** out of 1000 women |
| Cost | **$900** | **$0** |
|  | - **Strategy A** | - **Strategy B** |

b) **Now consider that you have an option of NO SCREENING (meaning no results, no strategy-related risk of miscarriage, and no costs). Based on the same pre-screening risk, which strategy would you prefer (tick only one box below)?**

|  | **Strategy selected in part a)** | **No Screening** |
| --- | --- | --- |
| Pre-screening risk of condition |  | **High**  *(Higher than 10 out of 1000 women)* |
| Time of results (weeks in pregnancy) |  | **No results** |
| Babies with an condition are missed |  | **No results** |
| Healthy babies have an inaccurate positive result |  | **No results** |
| Risk of miscarriage |  | **No additional risk** |
| Cost |  | **No cost** |
|  |  | - **No Screening** |

**Question 2 of 11**

a) Please imagine that you are considering prenatal screening and diagnosis. **You only have the following 2 strategies to choose from. Based on your pre-screening risk, which strategy would you prefer (tick only one box below)?**

|  | **Strategy A** | **Strategy B** |
| --- | --- | --- |
| Pre-screening risk of condition | **Low**  *(Less than 1 out of 1000 women)* | **Low**  *(Less than 1 out of 1000 women)* |
| Time of results (weeks in pregnancy) | **11** weeks | **17** weeks |
| Babies with an condition are missed | **10** out of 1000 babies | **100** out of 1000 babies |
| Healthy babies have an inaccurate positive result | **100** out of 1000 healthy babies | **20** out of 1000 healthy babies |
| Risk of miscarriage | **10** out of 1000 women | **0** out of 1000 women |
| Cost to you | **$0** | **$300** |
|  | - **Strategy A** | - **Strategy B** |

b) **Now consider that you have an option of NO SCREENING (meaning no results, no strategy-related risk of miscarriage, and no costs). Based on the same pre-screening risk, which strategy would you prefer (tick only one box below)?**

|  | **Strategy selected in part a)** | **No Screening** |
| --- | --- | --- |
| Pre-screening risk of condition |  | **Low**  *(Less than 1 out of 1000 women)* |
| Time of results (weeks in pregnancy) |  | **No results** |
| Babies with an condition are missed |  | **No results** |
| Healthy babies have an inaccurate positive result |  | **No results** |
| Risk of miscarriage |  | **No additional risk** |
| Cost to you |  | **No cost** |
|  |  | - **No Screening** |

**Question 3 of 11**

a) Please imagine that you are considering prenatal screening and diagnosis. **You only have the following 2 strategies to choose from. Based on your pre-screening risk, which strategy would you prefer (tick only one box below)?**

|  | **Strategy A** | **Strategy B** |
| --- | --- | --- |
| Pre-screening risk of condition | **Moderate**  *(Between 1 out of 1000 and 1 out of 100 women)* | **Moderate**  *(Between 1 out of 1000 and 1 out of 100 women)* |
| Time of results (weeks in pregnancy) | **17** weeks | **14** weeks |
| Babies with an condition are missed | **100** out of 1000 babies | **10** out of 1000 babies |
| Healthy babies have an inaccurate positive result | **100** out of 100 healthy babies | **0** out of 1000 healthy babies |
| Risk of miscarriage | **0** out of 1000 women | **5** out of 1000 women |
| Cost to you | **$300** | **$900** |
|  | - **Strategy A** | - **Strategy B** |

b) **Now consider that you have an option of NO SCREENING (meaning no results, no strategy-related risk of miscarriage, and no costs). Based on the same pre-screening risk, which strategy would you prefer (tick only one box below)?**

|  | **Strategy selected in part a)** | **No Screening** |
| --- | --- | --- |
| Pre-screening risk of condition |  | **Moderate**  *(Between 1 out of 1000 and 1 out of 100 women)* |
| Time of results (weeks in pregnancy) |  | **No results** |
| Babies with an condition are missed |  | **No results** |
| Healthy babies have an inaccurate positive result |  | **No results** |
| Risk of miscarriage |  | **No additional risk** |
| Cost to you |  | **No cost** |
|  |  | - **No Screening** |

**Question 4 of 11**

a) Please imagine that you are considering prenatal screening and diagnosis. **You only have the following 2 strategies to choose from. Based on your pre-screening risk, which strategy would you prefer (tick only one box below)?**

|  | **Strategy A** | **Strategy B** |
| --- | --- | --- |
| Pre-screening risk of condition | **High**  *(Higher than 10 out of 1000 women)* | **High**  *(Higher than 10 out of 1000 women)* |
| Time of results (weeks in pregnancy) | **14** weeks | **11** weeks |
| Babies with an condition are missed | **10** out of 1000 babies | **0** out of 1000 babies |
| Healthy babies have an inaccurate positive result | **20** out of 1000 healthy babies | **100** out of 1000 healthy babies |
| Risk of miscarriage | **0** out of 1000 women | **10** out of 1000 women |
| Cost to you | **$900** | **$300** |
|  | - **Strategy A** | - **Strategy B** |

b) **Now consider that you have an option of NO SCREENING (meaning no results, no strategy-related risk of miscarriage, and no costs). Based on the same pre-screening risk, which strategy would you prefer (tick only one box below)?**

|  | **Strategy selected in part a)** | **No Screening** |
| --- | --- | --- |
| Pre-screening risk of condition |  | **High**  *(Higher than 1 out of 100 women)* |
| Time of results (weeks in pregnancy) |  | **No results** |
| Babies with an condition are missed |  | **No results** |
| Healthy babies have an inaccurate positive result |  | **No results** |
| Risk of miscarriage |  | **No additional risk** |
| Cost to you |  | **No cost** |
|  |  | - **No Screening** |

**Question 5 of 11**

a) Please imagine that you are considering prenatal screening and diagnosis. **You only have the following 2 strategies to choose from. Based on your pre-screening risk, which strategy would you prefer (tick only one box below)?**

|  | **Strategy A** | **Strategy B** |
| --- | --- | --- |
| Pre-screening risk of condition | **Low**  *(Less than 1 out of 1000 women)* | **Low**  *(Less than 1 out of 1000 women)* |
| Time of results (weeks in pregnancy) | **14** weeks | **11** weeks |
| Babies with an condition are missed | **10** out of 1000 babies | **0** out of 1000 babies |
| Healthy babies have an inaccurate positive result | **0** out of 1000 healthy babies | **10** out of 1000 healthy babies |
| Risk of miscarriage | **0** out of 1000 women | **5** out of 1000 women |
| Cost to you | **$0** | **$900** |
|  | - **Strategy A** | - **Strategy B** |

b) **Now consider that you have an option of NO SCREENING (meaning no results, no strategy-related risk of miscarriage, and no costs). Based on the same pre-screening risk, which strategy would you prefer (tick only one box below)?**

|  | **Strategy selected in part a)** | **No Screening** |
| --- | --- | --- |
| Pre-screening risk of condition |  | **Low**  *(Less than 1 out of 1000 women)* |
| Time of results (weeks in pregnancy) |  | **No results** |
| Babies with an condition are missed |  | **No results** |
| Healthy babies have an inaccurate positive result |  | **No results** |
| Risk of miscarriage |  | **No additional risk** |
| Cost to you |  | **No cost** |
|  |  | - **No Screening** |

**Question 6 of 11**

a) Please imagine that you are considering prenatal screening and diagnosis. **You only have the following 2 strategies to choose from. Based on your pre-screening risk, which strategy would you prefer (tick only one box below)?**

|  | **Strategy A** | **Strategy B** |
| --- | --- | --- |
| Pre-screening risk of condition | **Moderate**  *(Between 1 out of 1000 and 1 out of 100 women)* | **Moderate**  *(Between 1 out of 1000 and 1 out of 100 women)* |
| Time of results (weeks in pregnancy) | **11** weeks | **17** weeks |
| Babies with an condition are missed | **0** out of 1000 babies | **100** out of 1000 babies |
| Healthy babies have an inaccurate positive result | **20** out of 1000 healthy babies | **0** out of 1000 healthy babies |
| Risk of miscarriage | **10** out of 1000 women | **5** out of 1000 women |
| Cost to you | **$900** | **$0** |
|  | - **Strategy A** | - **Strategy B** |

b) **Now consider that you have an option of NO SCREENING (meaning no results, no strategy-related risk of miscarriage, and no costs). Based on the same pre-screening risk, which strategy would you prefer (tick only one box below)?**

|  | **Strategy selected in part a)** | **No Screening** |
| --- | --- | --- |
| Pre-screening risk of condition |  | **Moderate**  *(Between 1 out of 1000 and 1 out of 100 women)* |
| Time of results (weeks in pregnancy) |  | **No results** |
| Babies with an condition are missed |  | **No results** |
| Healthy babies have an inaccurate positive result |  | **No results** |
| Risk of miscarriage |  | **No additional risk** |
| Cost to you |  | **No cost** |
|  |  | - **No Screening** |

**Question 7 of 11**

a) Please imagine that you are considering prenatal screening and diagnosis. **You only have the following 2 strategies to choose from. Based on your pre-screening risk, which strategy would you prefer (tick only one box below)?**

|  | **Strategy A** | **Strategy B** |
| --- | --- | --- |
| Pre-screening risk of condition | **High**  *(Higher than 10 out of 1000 women)* | **High**  *(Higher than 10 out of 1000 women)* |
| Time of results (weeks in pregnancy) | **14** weeks | **17** weeks |
| Babies with an condition are missed | **100** out of 1000 babies | **0** out of 1000 babies |
| Healthy babies have an inaccurate positive result | **100** out of 1000 healthy babies | **0** out of 1000 healthy babies |
| Risk of miscarriage | **5** out of 1000 women | **0** out of 1000 women |
| Cost to you | **$0** | **$300** |
|  | - **Strategy A** | - **Strategy B** |

b) **Now consider that you have an option of NO SCREENING (meaning no results, no strategy-related risk of miscarriage, and no costs). Based on the same pre-screening risk, which strategy would you prefer (tick only one box below)?**

|  | **Strategy selected in part a)** | **No Screening** |
| --- | --- | --- |
| Pre-screening risk of condition |  | **High**  *(Higher than 1 out of 100 women)* |
| Time of results (weeks in pregnancy) |  | **No results** |
| Babies with an condition are missed |  | **No results** |
| Healthy babies have an inaccurate positive result |  | **No results** |
| Risk of miscarriage |  | **No additional risk** |
| Cost to you |  | **No cost** |
|  |  | - **No Screening** |

**Question 8 of 11**

a) Please imagine that you are considering prenatal screening and diagnosis. **You only have the following 2 strategies to choose from. Based on your pre-screening risk, which strategy would you prefer (tick only one box below)?**

|  | **Strategy A** | **Strategy B** |
| --- | --- | --- |
| Pre-screening risk of condition | **Low**  *(Less than 1 out of 1000 women)* | **Low**  *(Less than 1 out of 1000 women)* |
| Time of results (weeks in pregnancy) | **17** weeks | **14** weeks |
| Babies with an condition are missed | **100** out of 1000 babies | **10** out of 1000 babies |
| Healthy babies have an inaccurate positive result | **0** out of 1000 healthy babies | **20** out of 1000 healthy babies |
| Risk of miscarriage | **10** out of 1000 women | **0** out of 1000 women |
| Cost to you | **$0** | **$300** |
|  | - **Strategy A** | - **Strategy B** |

b) **Now consider that you have an option of NO SCREENING (meaning no results, no strategy-related risk of miscarriage, and no costs). Based on the same pre-screening risk, which strategy would you prefer (tick only one box below)?**

|  | **Strategy selected in part a)** | **No Screening** |
| --- | --- | --- |
| Pre-screening risk of condition |  | **Low**  *(Less than 1 out of 1000 women)* |
| Time of results (weeks in pregnancy) |  | **No results** |
| Babies with an condition are missed |  | **No results** |
| Healthy babies have an inaccurate positive result |  | **No results** |
| Risk of miscarriage |  | **No additional risk** |
| Cost to you |  | **No cost** |
|  |  | - **No Screening** |

**Question 9 of 11**

a) Please imagine that you are considering prenatal screening and diagnosis. **You only have the following 2 strategies to choose from. Based on your pre-screening risk, which strategy would you prefer (tick only one box below)?**

|  | **Strategy A** | **Strategy B** |
| --- | --- | --- |
| Pre-screening risk of condition | **Moderate**  *(Between 1 out of 1000 and 1 out of 100 women)* | **Moderate**  *(Between 1 out of 1000 and 1 out of 100 women)* |
| Time of results (weeks in pregnancy) | **11** weeks | **14** weeks |
| Babies with an condition are missed | **0** out of 1000 babies | **100** out of 1000 babies |
| Healthy babies have an inaccurate positive result | **100** out of 1000 healthy babies | **20** out of 1000 healthy babies |
| Risk of miscarriage | **5** out of 1000 women | **10** out of 1000 women |
| Cost to you | **$300** | **$0** |
|  | - **Strategy A** | - **Strategy B** |

b) **Now consider that you have an option of NO SCREENING (meaning no results, no strategy-related risk of miscarriage, and no costs). Based on the same pre-screening risk, which strategy would you prefer (tick only one box below)?**

|  | **Strategy selected in part a)** | **No Screening** |
| --- | --- | --- |
| Pre-screening risk of condition |  | **Moderate**  *(Between 1 out of 1000 and 1 out of 100 women)* |
| Time of results (weeks in pregnancy) |  | **No results** |
| Babies with an condition are missed |  | **No results** |
| Healthy babies have an inaccurate positive result |  | **No results** |
| Risk of miscarriage |  | **No additional risk** |
| Cost to you |  | **No cost** |
|  |  | - **No Screening** |

**Question 10 of 11**

a) Please imagine that you are considering prenatal screening and diagnosis. **You only have the following 2 strategies to choose from. Based on your pre-screening risk, which strategy would you prefer (tick only one box below)?**

|  | **Strategy A** | **Strategy B** |
| --- | --- | --- |
| Pre-screening risk of condition | **High**  *(Higher than 10 out of 1000 women)* | **High**  *(Higher than 10 out of 1000 women)* |
| Time of results (weeks in pregnancy) | **17** weeks | **11** weeks |
| Babies with an condition are missed | **0** out of 1000 babies | **10** out of 1000 babies |
| Healthy babies have an inaccurate positive result | **20** out of 1000 healthy babies | **0** out of 1000 healthy babies |
| Risk of miscarriage | **5** out of 1000 women | **10** out of 1000 women |
| Cost to you | **$300** | **$900** |
|  | - **Strategy A** | - **Strategy B** |

b) **Now consider that you have an option of NO SCREENING (meaning no results, no strategy-related risk of miscarriage, and no costs). Based on the same pre-screening risk, which strategy would you prefer (tick only one box below)?**

|  | **Strategy selected in part a)** | **No Screening** |
| --- | --- | --- |
| Pre-screening risk of condition |  | **High**  *(Higher than 1 out of 100 women)* |
| Time of results (weeks in pregnancy) |  | **No results** |
| Babies with an condition are missed |  | **No results** |
| Healthy babies have an inaccurate positive result |  | **No results** |
| Risk of miscarriage |  | **No additional risk** |
| Cost to you |  | **No cost** |
|  |  | - **No Screening** |

**Question 11 of 11**

a) Please imagine that you are considering prenatal screening and diagnosis. **You only have the following 2 strategies to choose from. Based on your pre-screening risk, which strategy would you prefer (tick only one box below)?**

|  | **Strategy A** | **Strategy B** |
| --- | --- | --- |
| Pre-screening risk of condition | **Low**  *(Less than 1 out of 1000 women)* | **Low**  *(Less than 1 out of 1000 women)* |
| Time of results (weeks in pregnancy) | **11** weeks | **17** weeks |
| Babies with an condition are missed | **0** out of 1000 babies | **100** out of 1000 babies |
| Healthy babies have an inaccurate positive result | **0** out of 1000 healthy babies | **20** out of 1000 healthy babies |
| Risk of miscarriage | **0** out of 1000 women | **5** out of 1000 women |
| Cost to you | **$300** | **$300** |
|  | - **Strategy A** | - **Strategy B** |

b) **Now consider that you have an option of NO SCREENING (meaning no results, no strategy-related risk of miscarriage, and no costs). Based on the same pre-screening risk, which strategy would you prefer (tick only one box below)?**

|  | **Strategy selected in part a)** | **No Screening** |
| --- | --- | --- |
| Pre-screening risk of condition |  | **Low**  *(Less than 1 out of 1000 women)* |
| Time of results (weeks in pregnancy) |  | **No results** |
| Babies with an condition are missed |  | **No results** |
| Healthy babies have an inaccurate positive result |  | **No results** |
| Risk of miscarriage |  | **No additional risk** |
| Cost to you |  | **No cost** |
|  |  | - **No Screening** |

### SECTION B: About Me

This section has 17 questions that ask about you and your experiences with pregnancy. Please answer the questions on the following pages.

1. What is your age category?

___ Under 25 years

___ 25 to 29 years

___ 30 to 34 years

___ 35 to 39 years

___ 40 years to 49 years

___ 50 years to 59 years

___ 60 years or over

___ Prefer not to say

1. Were you born in Canada?

___ Yes

___ No

___ Prefer not to say

1. Which of the following best describes your ethnicity? *Please select all that apply.*

___ Aboriginal (e.g., First Nations, Métis, Inuit, etc.)

___ African (e.g., Haitian, Nigerian, etc.)

___ East Asian (e.g., Chinese, Korean, Japanese, Malaysian, etc.)

___ European (e.g., British, French, German, Czech, Polish, etc.)

___ Latin American (e.g., Mexican, Colombian, etc.)

___ South Asian (e.g., East Indian, Pakistani, etc.)

___ West Asian (e.g., Persian, Turk, Arab, Armenian, etc.)

___ Other

___ Don’t know

___ Prefer not to say

##### Do you have a religious faith?

___ Yes

___ No

___ Prefer not to say

1. What is the highest level of education that you have completed? *Please select one*

___ Primary School

___ High School

___ College or Technical/Trade

___ University Degree

___ Post-Graduate Degree or Professional Designation

___ Other

___ Prefer not to say

1. What is your current employment status? *Please select one*

___ Student

___ Employed, full time

___ Employed, part time

___ Unemployed

___ Retired

___ Other, please specify: ________________________

___ Prefer not to say

1. This question asks about your household income. This information is very private (and will be kept confidential). Please indicate the best estimate of your total annual household income, before taxes, including all household members:

___ No income

___ Under $35,000

___ $35,000 to $74,999

___ $75,000 to $109,999

___ Over $110,000

___ Don’t know

___ Prefer not to say

1. What is your marital status? *Please select one*

___ Single (never legally married)

___ Married (and not separated)

___ Common-law

___ Separated

___ Divorced

___ Widowed

___ Prefer not to say

##### Do you or your partner have any children?

___ Yes

___ No **(**Go to #11)**

___ Prefer not to say **(**Go to #11)**

1. Do you or your partner have a child with a chromosomal condition (e.g., Down syndrome)?

___ Yes

___ No

___ Prefer not to say

1. Do you know anyone with a child with a chromosomal condition (e.g., Down syndrome)?

___ Yes

___ No

___ Prefer not to say

1. Have you or your partner ever had any problems conceiving a child (been unable to become pregnant or been clinically diagnosed with infertility)?

___ Yes

___ No

___ Not applicable

___ Don’t know

___ Prefer not to say

1. Have you ever had or plan to have prenatal screening for chromosomal conditions (e.g., Down syndrome) in this pregnancy?

___ Have screened

___ Plan to screen

___ Do not plan to screen

___ Don’t know

___ Prefer not to say

1. Have you ever been pregnant previously?

___ Yes

___ No **(**Go to #16)**

___ Don’t know **(**Go to #16)**

___ Prefer not to say **(**Go to #16)**

1. Have you ever had prenatal screening for chromosomal conditions (e.g., Down syndrome) in any previous pregnancies?

___ Have had previous screening

___ Have not had previous screening

___ Don’t know

___ Don’t remember

___ Prefer not to say

1. Have you ever had a miscarriage?

___ Yes

___ No

___ Prefer not to say

1. Would you consider abortion if your baby had a confirmed chromosomal condition?

___ Only if the baby had a life threatening condition

___ Only if the baby had an intellectual disability

___ Yes, in both cases

___ No

___ Don’t know

___ Prefer not to say

SECTION C: Final Thoughts and Comments

Please use the space below for any comments you may have about:

1. This survey
2. Any other factors you think are important about prenatal screening and diagnosis

THANK YOU FOR HELPING US WITH THIS RESEARCH
