# Supplementary material for: The impact of psychological distance on preferences for prenatal screening and diagnosis for chromosomal abnormalities: A hierarchical Bayes analysis of a discrete choice experiment
Source: PLoS One. 2025 May 23;20(5):e0324370. doi: 10.1371/journal.pone.0324370 (PMC12101744; doi:10.1371/journal.pone.0324370)
Supplement: S2 File — (DOCX) [file pone.0324370.s012.docx]

**Sample size calculation**

According to Johnson and Orme^[[1]](#footnote-1)^, one rule of thumb for the minimum sample size for DCEs is:

n≥500c/ta

where:

n is the number of respondents,

t is the number of tasks,

a is number of alternatives per task (not including the none alternative),

c is the largest number of levels for any one attribute

1. Johnson R, Orme B. Sample size issues for conjoint analysis. In: Orme B (editor). Getting started with conjoint analysis: strategies for product design and pricing research. Madison: Research Publishers; 2010. p. 57–66 [↑](#footnote-ref-1)
