# Supplementary material for: The impact of psychological distance on preferences for prenatal screening and diagnosis for chromosomal abnormalities: A hierarchical Bayes analysis of a discrete choice experiment
Source: PLoS One. 2025 May 23;20(5):e0324370. doi: 10.1371/journal.pone.0324370 (PMC12101744; doi:10.1371/journal.pone.0324370)
Supplement: S3 File — (DOCX) [file pone.0324370.s013.docx]

**Utility function and mWTP calculation**

The utility function used in our study can be described as:

U=$ASC+\sum\alpha_{ijz}$+$\beta_{t}. T_{i}+ \delta. C_{i}$

And mWTP for strategy i was calculated based on the following formula:

$${mWTP}_{i}=\frac{\sum\left( \alpha_{ijz}-\alpha_{1jz} \right)+\beta_{t}(T_{i}-T_{1})}{\delta}$$

Where:

ASC: Alternative specific constant

​ $\alpha_{ijz}$is the coefficient of the level z of the effect-coded attribute j associated with strategy i.

​$\alpha_{1jz}$ is the coefficient of the level j of the effect-coded attribute j associated with the base case.

$\beta_{t}$is the coefficient for the time to results.

Ti and T1 are the time to results associated with strategy i and base case.

$\delta$ is the coefficient of cost.

Ci is the out of pocket cost for strategy i.
